# Whole genome sequencing refines stratification and therapy of patients with clear cell renal cell carcinoma

**DOI:** 10.21203/rs.3.rs-3675752/v1

**Published:** 2023-12-06

**Authors:** Richard Houlston, Richard Culliford, Sam Lawrence, Charlie Mills, Zayd Tippu, Daniel Chubb, Alex Cornish, Lisa Browining, Ben Kinnersley, Robert Bentham, Amit Sud, Husayn Pallikonda, Anna Frangou, Andreas Gruber, Kevin Litchfield, David Wedge, James Larkin, Samra Turajlic

**Affiliations:** The Institute of Cancer Research; The Institute of Cancer Research; The Institute of Cancer Research; The Institute of Cancer Research; The Francis Crick Institute; Institute of Cancer Research; The Institute of Cancer Research; University of Oxford; UCL Cancer Institute; The Institute of Cancer Research; The Institute of Cancer Research; The Francis Crick Institute; University of Oxford; University of Konstanz; University College London Cancer Institute; University of Manchester; The Royal Marsden Hospital; Francis Crick Institute

## Abstract

Clear cell renal cell carcinoma (ccRCC) is the most common form of kidney cancer, but a comprehensive description of its genomic landscape is lacking. We report the whole genome sequencing of 778 ccRCC patients enrolled in the 100,000 Genomes Project, providing the most detailed somatic mutational landscape to date. We identify new driver genes, which as well as emphasising the major role of epigenetic regulation in ccRCC highlight additional biological pathways extending opportunities for drug repurposing. Genomic characterisation identified patients with divergent clinical outcome; higher number of structural copy number alterations associated with poorer prognosis, whereas VHL mutations were independently associated with a better prognosis. The twin observations that higher T-cell infiltration is associated with better outcome and that genetically predicted immune evasion is not common supports the rationale for immunotherapy. These findings should inform personalised surveillance and treatment strategies for ccRCC patients.

## INTRODUCTION

Renal cell carcinoma (RCC) is an increasing global health problem with 431,000 new diagnoses each year, set to increase to 666,000 by 2040^1^. Around 75% of RCCs are clear cell RCC (ccRCC) tumours. These cancers have a variable clinical course and while 75–80% of patients present with apparently localised disease and are offered curative intent treatment 30% will subsequently relapse^2^. There is therefore a pressing need for more accurate risk stratification, to guide clinical decisions relative to therapy and surveillance.

While therapeutic advances in the treatment of metastatic ccRCC have been made with the advent of antiangiogenic targeted therapies and immune checkpoint inhibitors (iCPi) only a fraction of patients experience durable clinical benefit. Mixed outcomes of adjuvant PD1-therapies demonstrates that clinical biomarkers fail to reconcile the variable disease course following surgery^3–5^.

The need to understand ccRCC biology to inform development of novel therapies and better predict patient outcomes has been a major motivation in sequencing studies. While these projects have identified recurrent gene mutations and chromosomal rearrangements analyses have primarily been based on whole-exome sequencing or panel testing of cancer-associated genes, hence the full complement of drivers is incomplete. Correspondingly, studies of the relationship between clinical parameters and genomic alterations have been limited^6–9^. To advance our understanding of ccRCC we analysed whole genome sequencing (WGS) data from 778 ccRCC patients recruited to the UK Genomics England (Gel) 100,000 Genomes Project (100kGP)^10^.

## RESULTS

### The Gel cohort

**Figure 1:**
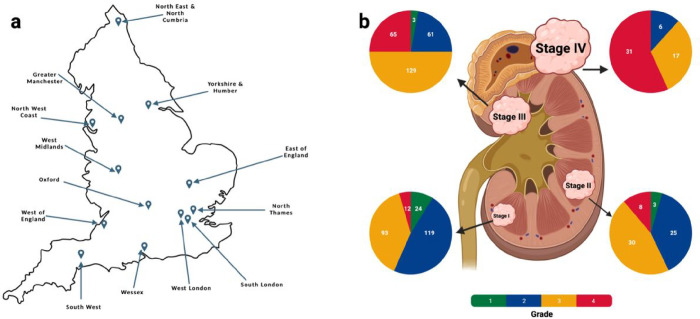
Overview of the Gel cohort of ccRCC patients. **(a)** The location of the 13 Genomic Medicine Centers (GMCs) across England from which patients were recruited; **(b)** The breakdown of the cohort by tumour grade and stage. Figure created using BioRender.

The analysed cohort (100kGP, release v14) comprised tumour-normal (T/N) sample pairs from 778 patients (mean age 63 years, range 25–88 years) with primary ccRCC recruited to 100kGP through 13 Genomic Medicine Centres across England (Fig. 1). Comprehensive clinico-pathology information on the patients is provided in **Supplementary Table 1.** We restricted our WGS analysis to samples with high-quality data from PCR-free, flash-frozen fresh tumour samples (**Supplementary Methods**). For 29 of the patients WGS data on multi-regional sampling of tumours was available (2–4 samples per tumour, 94 samples in total). In addition to using variant calls from the 100kGP analysis pipeline we: (i) removed alignment bias introduced by ISAAC soft clipping of semi-aligned reads^11^; (ii) called tumour copy number using Battenberg^12^; (iii) called structural variants (SVs) from a consensus of Manta^13^, LUMPY^14^, and DELLY^15^; (iv) removed insertion-deletions (indels) within 10 base pairs (bp) of a common germline indel. Complete details on sample curation, somatic variant calling, and annotation of mutations are provided in the **Supplementary Methods**.

Restricting our analysis to WGS data on one sample per patient (**Supplementary Methods**), we identified 4,267,943 single nucleotide variants (SNVs), 699,100 indels, and 19,756 chromosomal rearrangements or structural variants (**Supplementary Table 2**). While the median tumour mutational burden was 1.88/Mb, three tumours displayed a hypermutated phenotype: *i.e*., excessively high SNV/indel mutation burden (maximal SNV/Mb = 33.65, maximal indel/Mb = 21.77). Twenty-two of the patients (2.8%) were carriers of pathogenic germline variants in one of the well-established RCC susceptibility genes and 10 (1.2%) were carriers of a variant in another cancer susceptibility gene (**Supplementary Table 3**).

### Driver mutations

**Figure 2:**
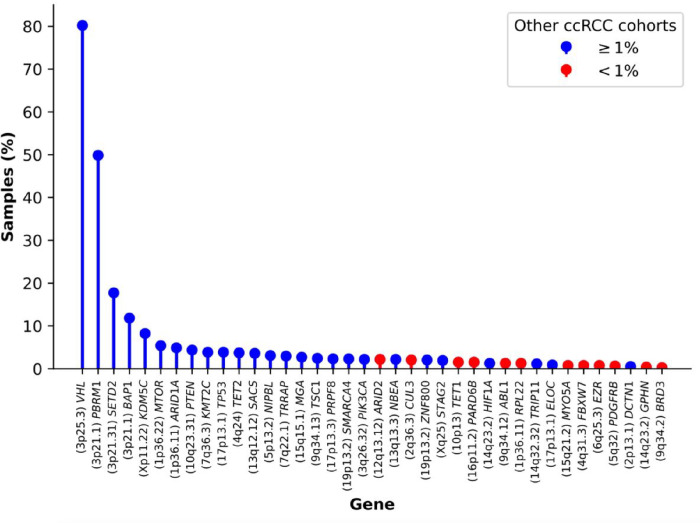
Frequency of nonsynonymous mutations in driver genes. The colour scheme indicates whether the mutational frequency of a driver gene is reported as being above (blue) or below (red) 1% in other ccRCC cohorts.

Protein-coding driver gene identification at the base pair level was performed using IntOGen^16^, which incorporates seven complementary algorithms. A total of 38 genes were identified as driver genes, including 25 well-recognized drivers and 13 which either had not been reported previously or have frequencies < 1% in landmark genomics studies of ccRCC^17–21^ (Fig. 2). Of the mutations annotated by AlphaMissense^22^, 72.4% of driver gene missense SNVs (436/602) were predicted to be pathogenic as compared to 29.8% (11,112/37,297) of the missense SNVs in non-driver genes (*P* = 4.2 × 10^−112^). The major known ccRCC driver genes were mutated at close to reported frequencies^17–19,21,23–25^: *VHL* (80.2%), *PBRM1* (49.9%), *SETD2* (17.7%) and *BAP1* (11.8%). Subclonal drivers, such as TSC1 were, however seen with lower frequency, likely due to the use of a single biopsy rather than multi-regional sampling^8^ (**Supplementary Table 4**). All of the novel coding drivers were detected at low frequencies, as expected given the scale of previous exome sequencing studies (0.3–2.4%, **Supplementary Table 4**; **Supplementary Fig. 1**). Mutations in these novel drivers were frequently accompanied by loss of heterozygosity (LOH) (see “Recurrent structural and copy number alterations”), implying loss of function (**Supplementary Fig. 2**).

**Figure 3:**
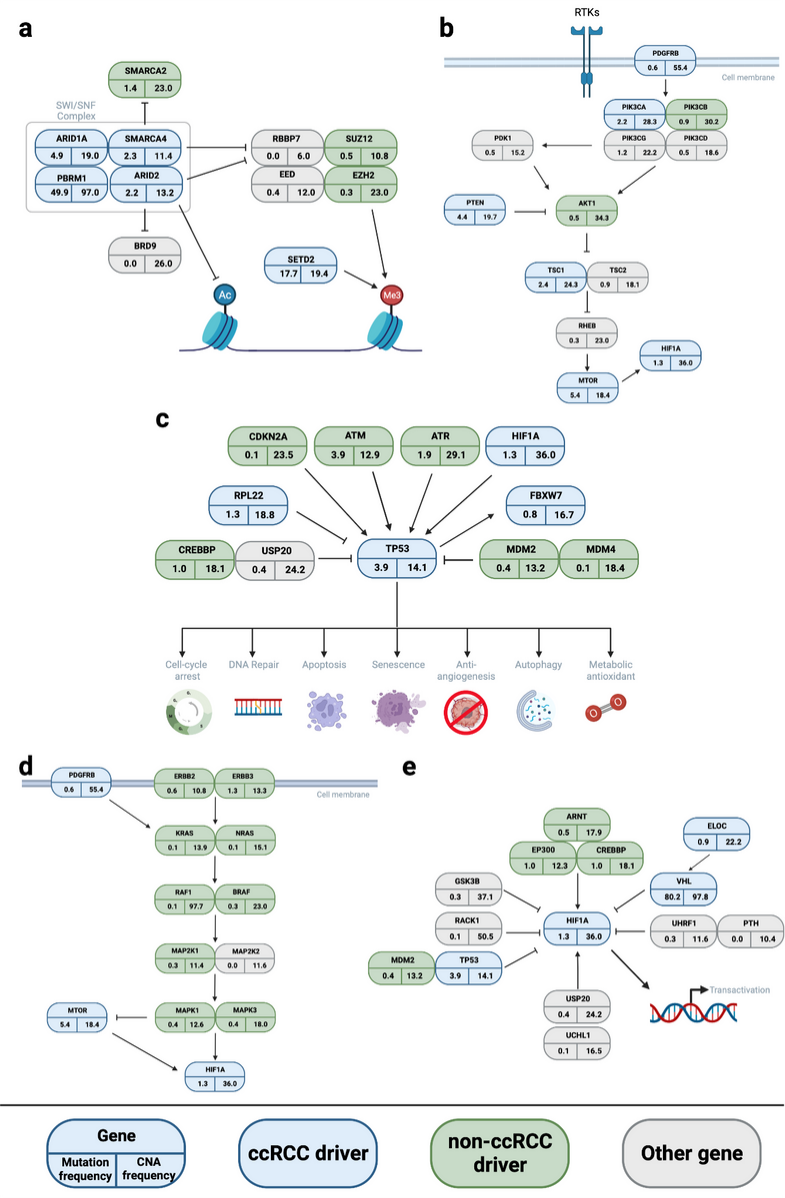
Biological pathways in ccRCC. **(a)** The *SWI/SNF* pathway; **(b)** The *MAPK* signalling pathway; **(c)**The *TP53* pathway; **(d)**The *RAS/ERK* and hypoxia pathway; **(e)**The *VHL/HIF1A* pathway. Driver genes identified shown in blue, non-ccRCC driver genes in green and other pathway genes in grey. The number in the bottom left is the nonsynonymous mutational frequency and the number in the bottom right the copy number alteration (CNA) frequency. RTK, Receptor Tyrosine Kinase. Figure created using BioRender.

Novel drivers emphasise the central importance of epigenetic modification in the development of ccRCC through SWI/SNF mediated chromatin remodelling (*BRD3, ARID2*), histone deubiquitination (*CUL3, FBXW7*) as well as the role of methylcytosine dioxygenase activity (*TET1*). Biological mechanisms highlighted by the new drivers are shown in Fig. 3 (**Supplementary Table 4**). These included new vessel formation (*PDGFR-β*), ribosomal activity (*RPL22*), cytoskeletal interactions (*EZR, GPHN, NBEA*), cell division and cell polarisation (*PARD6B*). Mutations of *ARID2, CUL3, TET1*, *FBXW7, EZR* and *GPHN* were frequently accompanied by copy number loss consistent with their predicted/documented tumour suppressor roles (**Supplementary Table 4**). As previously reported^26^, mutations in *VHL* and *ELOC*, which both play a key role in oxygen sensing and degradation of hypoxia-inducible factors, were mutually exclusive (*Q* = 0.002). Co-occurrence analysis also supported mutually exclusive relationships: *BAP1* and *PBRM1* (*Q* = 4.36 × 10^−12^), SWI/SWF mediated chromatin modifiers *PBRM1* and *ARID2* (*Q* = 0.04), and *SETD2* and *BAP1* (*Q* = 0.049). In contrast, mutations of *ARID2* and *ELOC* (*Q* = 0.001), and *PBRM1* and *SETD2* (*Q* = 1.33 × 10^−5^), tended to co-occur (**Supplementary Table 5**). In five tumours *BAP1* mutations were the sole driver.

To identify non-coding drivers in gene promoters, untranslated and non-canonical splice regions, we used OncodriveFML^27^, ActiveDriverWGS^28^, and negative binomial regression adjusting for trinucleotide mutational context (**Supplementary Methods**). The only mutated region, displaying consistent evidence of positive selection, was the *TERT* promoter region. This association was primarily driven by the canonical mutations 5:1295113G > A and 5:1295135G > A, both of which were reported as recurrent in TRACERx^29^ and have been documented to be early drivers for bladder cancer^30,31^ **(Supplementary Table 6).**

Systematic analyses of cancer genomes provide an opportunity of estimating the number of patients eligible for a targeted therapy and identify opportunities for drug repurposing. We assessed the clinical actionability of driver gene mutations by referencing OncoKB Knowledge Base^32^ (version 3.11), and found 93 unique alterations were targetable (OncoKB Level 1–4), and were all at least Level 4 (compelling biological evidence supporting the biomarker being predictive of drug response). We also examined COSMIC Mutation Actionability in Precision Oncology^33^ database highlighting an additional 717 unique alterations which are potentially targetable (**Supplementary Fig. 3, Supplementary Table 7**).

### Recurrent structural and copy number alterations

**Figure 4:**
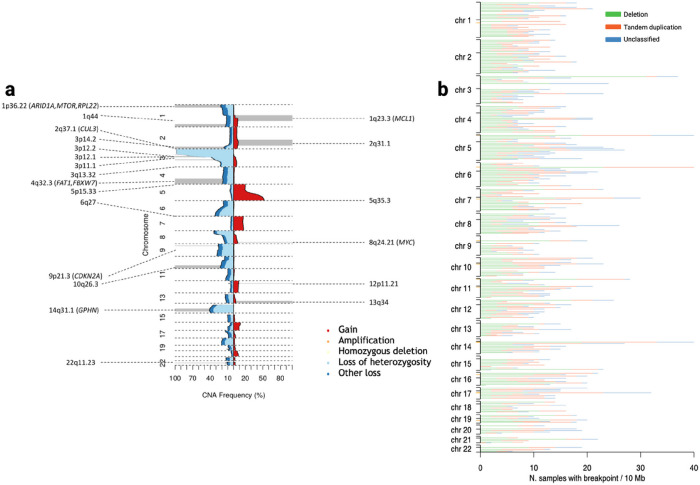
Copy number alterations and structural variants. **(a)** Frequency of copy number alterations across the ccRCC cohort. Copy number losses are coded in blue shades and copy number gains shown in red. The focal copy number alterations, as identified by GISTIC, are annotated along with predicted target gene; **(b)** The distribution of structural variants across the ccRCC cohort. Structural variants are classified as deletions, tandem duplications or unclassified structural variants. The black ticks on the y axis correspond to the chromosome start, centromere and end position while the orange ticks represent identified structural variant hotspot regions.

In addition to the previously reported common 3p loss, 5q gain and 14q loss^7,29^ we identified 25 other arm-level alterations that occurred more frequently than expected (Fig. 4, **Supplementary Table 8**). We used GISTIC2^36^ to identify genomic regions recurrently affected by focal amplifications and deletions (*Q* < 0.05; Fig. 4; **Supplementary Table 9**). Aside from the previously reported CNAs^7^, including del9p21.3 (*CDKN2A*), del3p12.2 (*GBE1*), amp5q35.3 (*SQSTM1*) and amp 8q24.21 (*MYC*) we identified four novel CNAs: amp2q31.1 (*ACVR2A, CASP8, NFE2L2, PMS1, SF3B1*), amp13q34 (*ERCC5*), amp12p11.21 and del22q11.23. Of the genes implicated by these novel focal amplifications, *NFE2L2* and *SF3B1* are documented to be oncogenic^37–40^. Pan-chromosome, 13.2% of tumours showed evidence of chromothripsis. Between chromosomes 3p and 5q, chromothripsis was only detected at low frequency (2.6%) and the rate of unbalanced translocations was 4.0%, in contrast to some^29^ but not all previous reports^34^. 16.6% of the tumours displayed whole genome duplication (WGD), a finding almost identical to the 15% reported by TracerX^35^ (**Supplementary Table 2**).

We identified 37 hotspots of recurrent simple SVs (FDR < 0.05) by piecewise constant fitting adjusting for local genomic features known to influence rearrangement density (chromatin accessibility, repeated elements, GC content, replication timing, gene density and expression). Fragile sites are prone to rearrangement (possibly due to replication error) and tend to co-occur with large, late-replicating genes. SVs occurring at fragile sites are hence likely to be the consequence of mechanistic rather than selective factors. After excluding 10 SV hotspots mapping to potential fragile sites, we identified 27 SV hotspots (Fig. 4, **Supplementary Table 10**). We identified a total of 66 breakpoints within 5p15.33, spanning *TERT*. These included, a deletion breakpoint and an unclassified event 2kb downstream of the *TERT* promoter, and tandem duplications overlapping *TERT* (n = 5). In tumours from the 34 patients with a *TERT* 5’UTR mutations, there were no overlapping unclassified/tandem-duplication events or a SV deletion/unclassified promoter breakpoints; an observation consistent with earlier findings^29^.

### Mutational signatures

**Figure 5:**
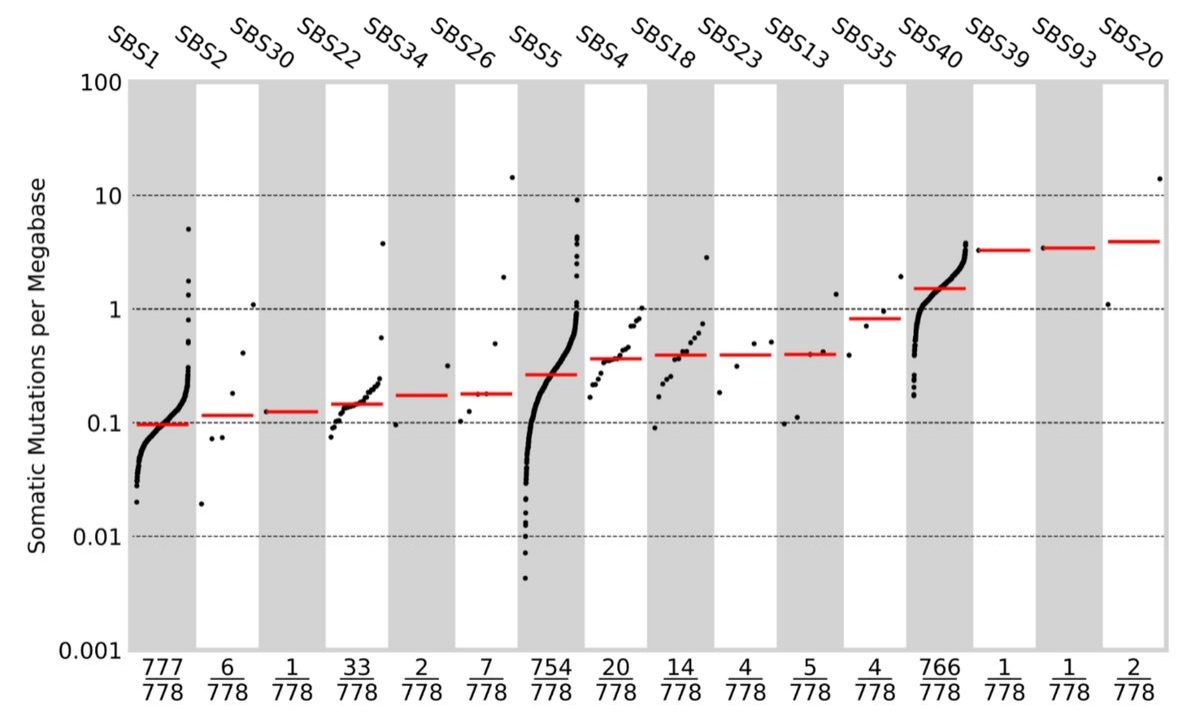
Mutational signatures. The mutational burden of single base substitution signatures.

To gain insight into mutational processes in ccRCC, we extracted single-base substitution (SBS), double-base-substitution (DBS) and indel (ID) signatures *de novo* and related those to known COSMIC signatures (v3.2) using SigProfilerExtractor^41,42^. In the majority of cancers, single base substitutions could be assigned to signatures SBS5/SBS40 and SBS1 (nomenclature as per COSMIC) resulting from clock-like mutagenic processes (Fig. 5, **Supplementary Fig. 4, Supplementary Table 11**). Other signatures recovered with known specific underlying aetiology include those associated with oxidative damage (SBS18), defective base excision repair (SBS30), APOBEC (SBS2, SBS13), tobacco smoking (SBS4, DBS2, ID3) and aristolochic acid (SBS22). While the incidence of renal cancer has been linked to aristolochic acid exposure in residents of Danube river countries^43^, 88% of patients with SBS22 tumour activity in the Gel cohort were self-reported to be white British. SBS31 and SBS35 have been attributable to platinum chemotherapy. We recovered SBS35 in four cases, none of which were reported to have a past history of platinum chemotherapy. In contrast the tumours from five patients, which had a past history of a non-RCC cancer and had received carboplatin or oxaliplatin did not display SBS31 or SBS35. To complement SigProfilerExtractor we searched for mutational signatures associated with defective mismatch repair (dMMR) and defective homologous recombination (dHR) using mSINGs^44^ and HRdetect^45^. Three cases displayed evidence of dMMR, of which two harboured *MLH1* somatic mutations accompanied by LOH, but none carried germline pathogenic MMR variants. Two of the cases had mutations assigned to signatures associated with dMMR (SBS20, SBS26). No case showed evidence of dHR. Considering mutational signature activity between clonal and subclonal mutations we found no significant enrichment or depletion of any SBS signatures between clonal and subclonal mutations.

### Ordering of mutational events

**Figure 6:**
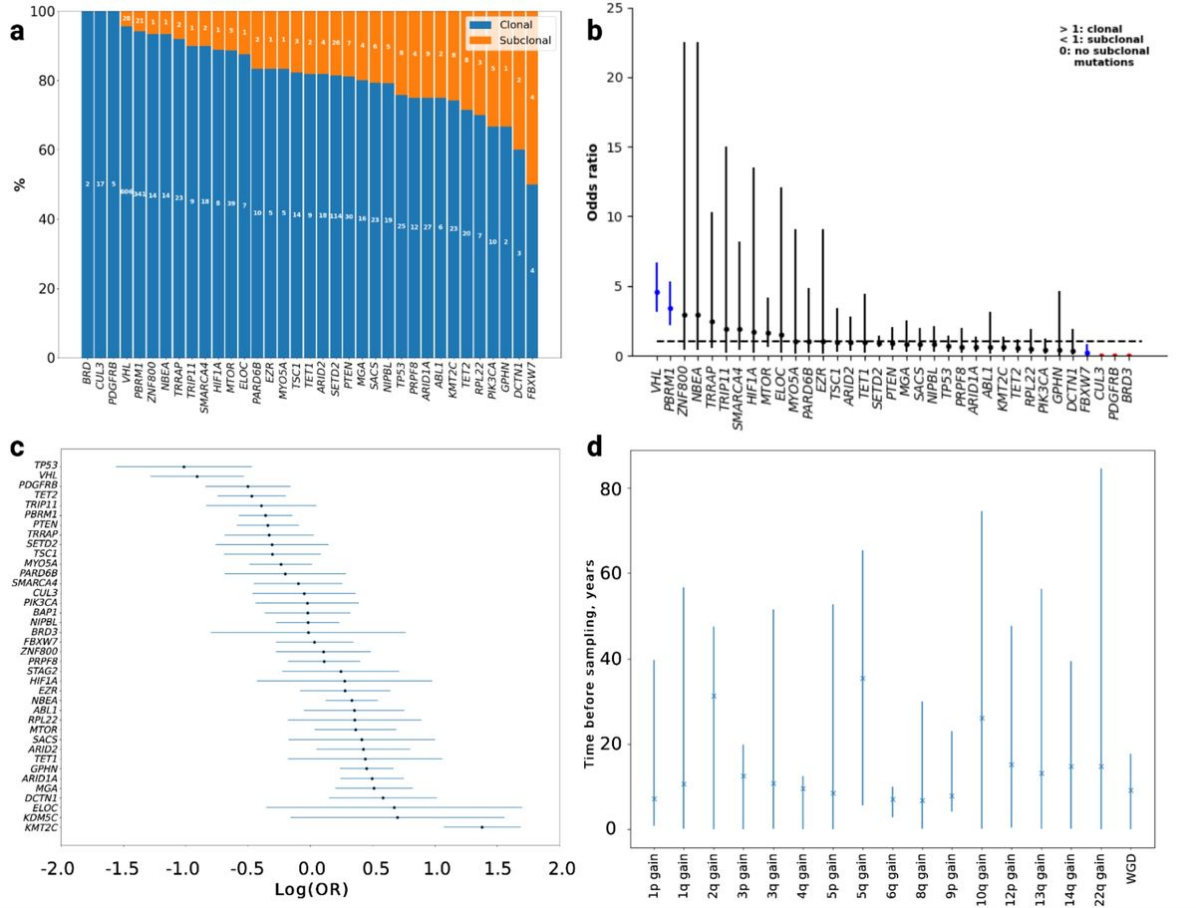
Mutational timing. **(a)** The proportion of clonal and subclonal nonsynonymous mutations in driver genes; **(b)**Odds ratio (OR) with 95% confidence intervals that a mutation in a driver gene is clonal; OR >1.0 indicates a mutation is more likely to be clonal. The genes in blue are significantly more likely to be clonal or subclonal. Genes in red have no subclonal mutations; **(c)** The relative ordering of mutations in driver genes; **(d)** Estimates of the real time at which copy number gains occur during tumour evolution.

Using PhylogicNDT^46^ in conjunction with MutationTimeR^47^, we reconstructed the chronological ordering of focal CNAs and driver mutations. Across all tumours gain of 5q were consistently earlier alterations. As expected, mutations in *VHL, PBRM1, SETD2* and *BAP1* were predicted to be early events, generally occurring before corresponding CNAs. In contrast, mutations in *KMT2C, ARID1A* and *HIF1A* were late events (Fig. 6). Estimating the chronological timing of CNAs under varying mutational rates and tumour initiation (**Supplementary Methods**) implies WGD occurred on average 9.2 years before tumour sampling and gain of 5q, 35.5 years before sampling (Fig. 6). Moreover, the estimated lead time of 5q gain and WGD were both correlated with age at presentation (adjusting for h grade and stage *P* = 9.4 × 10^−13^ and 0.02 respectively).

### Immune evasion

**Figure 7:**
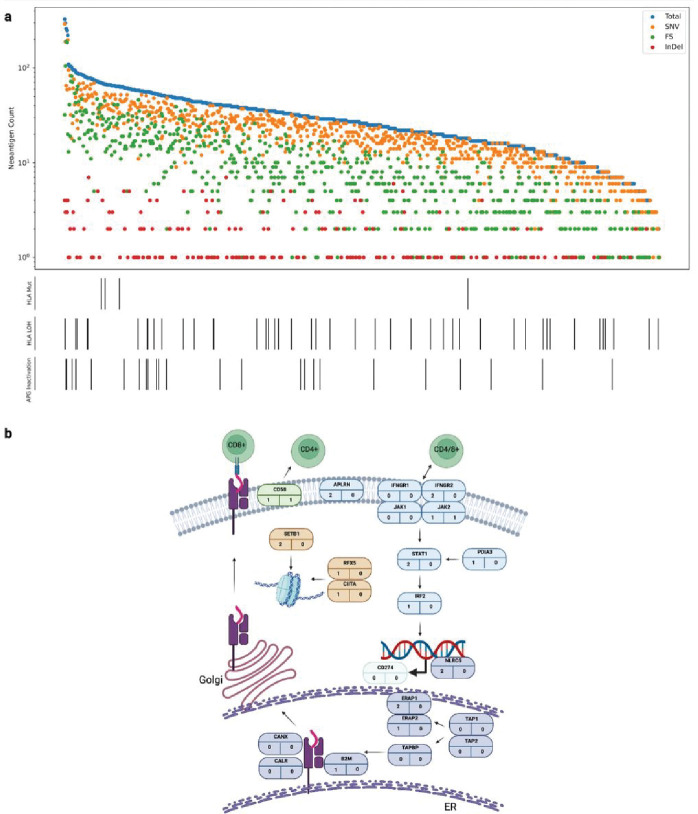
Immune landscape of ccRCC. **(a)** Neoantigen burden and immune escape mutations. Lower bars show antigen processing genes (APG) and HLA alterations present in each cancer; **(b)** Somatic mutations in each of the antigen presentation pathway genes. The number in the bottom left is the truncating mutation count and the number in the bottom right the number of biallelic nonsynonymous mutations. APGs in purple, IFN-γ pathway genes in blue, epigenetic modifier genes in brown, *CD274* comprises the PD-L1 receptor and CD58 receptor is encoded by *CD58*. Figure created using BioRender.

Using pVAC-Seq^48^, we predicted 24,893 class I neoantigens across the 778 tumours (1–327 per tumour, median 26), resulting from: 66.5% missense mutations, 32.0% frameshift variants, 1.3% inframe deletions and 0.25% inframe insertions (Fig. 7). As expected, TMB was positively associated with tumour neo-antigen count (TNC) (OR = 1.21, 95% CI: 1.18–1.23; **Supplementary Table 12**). Examining evidence of immune evasion we considered (LOH) or mutation of HLA class I genes (*HLA-A, HLA-B, HLA-C*) and immune escape genes (**Supplementary Methods, Supplementary Table 13**). Using LOHHLA, we detected LOH of HLA in only 5.9% of tumours. It has been reported that LOH on HLA class I genes and 9p21 loss tend to co-occur^49^, suggesting a potential mechanism for immune escape. However, after adjusting for stage and grade (correlated with both LOH of HLA and 9p21 loss, **Supplementary Table 14**) the correlation was not significant (OR = 1.32, 95% CI: 0.58–2.99; **Supplementary Table 15**). Similarly, nonsynonymous mutations of HLA genes were rare (0.5%). An inactivating mutation in at least one of the 22 antigen presenting genes^50,51^ (APG) was seen in only 3.1% (24/778) of tumours. None of the APGs displayed a propensity for mutation. Collectively, on the basis of alteration of these escape pathways only 9.0% (70/778) of tumours were predicted to exhibit some form of genetically-driven immune evasion.

### Clinico-pathological relationships

Increased tumour grade was associated with necrosis (*P* = 9.4 × 10^−15^), increased TMB (P = 0.001) and mutational sub-clonality (*P* = 0.02). High SV count (*P* = 1.5 × 10^−11^), WGD (*P* = 1.2 × 10^−6^) and weighted genome instability index (*P* = 1.7 × 10^−11^) were associated with higher tumour grade. Increased T-cell receptor alpha (TCRA) T-cell fraction^52^ (*i.e.* fraction of T-cells present in the WGS sample), reflecting immune infiltration, was associated with increased tumour grade (*P* = 4.0 × 10^−4^). Consistent with previous literature^53^, tumours with mutations in *BAP1* and *TP53* were more likely to present as high grade (*P* = 1.8 × 10^−5^, P = 2.1 × 10^−6^, respectively). Mutation of driver genes was associated with tumour stage (**Supplementary Table 14**).

**Figure 8:**
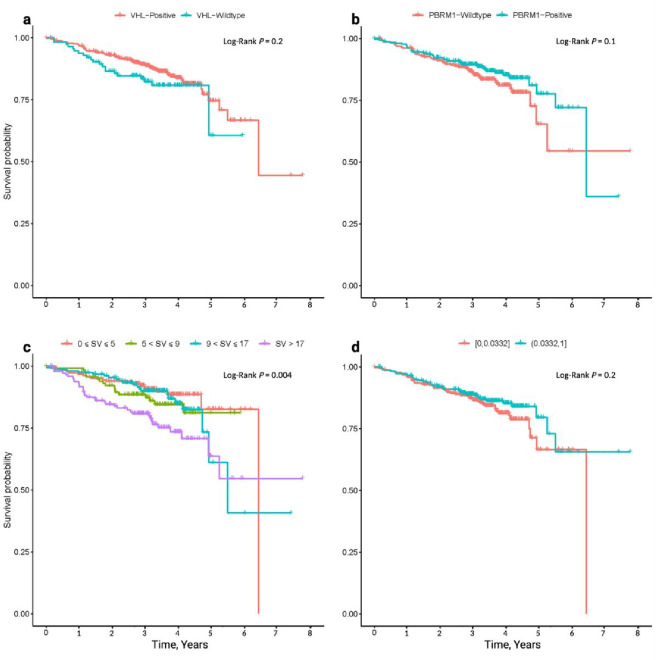
Kaplan Meier survival curves of overall survival. Relationship between OS and **(a)** *VHL* mutation status; **(b)** *PBRM1* mutation status; **(c)** Structural variant (SV) count; **(d)** TCRA T-cell fraction.

After excluding patients with missing follow up information we examined the relationship between genomic features and overall survival (OS) in 605 patients (**Supplementary Tables 1, 15, 16, Supplementary Figs. 5 and 6**.). Strong predictors of OS were age, grade and stage (Log-rank *P* = 6.3 × 10^−4^, *P* = 8 × 10^−15^ and *P* = 3 × 10^−15^ respectively). After adjusting for co-variants using Cox regression increased OS was associated with *VHL* (Hazard Ratio (HR) = 0.60, 95% CI: 0.36–0.98) and *PBRM1* (HR = 0.64, 95% CI: 0.42–0.97) mutation status (Fig. 8). Given the co-occurrence of *VHL* and *PBRM1* mutations, 84%, 325/388 of *PBRM1*-positive tumours were also *VHL* mutated), after adjusting for *VHL* status, *PBRM1* mutational status did not show an independent relationship with OS (HR = 0.68, 95% CI: 0.44–1.03). Aside from *VHL*, mutation of none of the other driver genes showed an independent association with OS; acknowledging we had limited statistical power to demonstrate a relationship with less frequently mutated genes. After adjusting for *VHL* status, higher SV count was, however, associated with worse OS (HR = 1.01, 95% CI: 1.00–1.10). We also observed that four specific copy number gains were associated with better OS. While we found no association between OS and either neoantigen burden or immune escape a higher TCRA T-cell fraction was associated with a better OS (HR = 0.65, 95% CI: 0.43–0.99; Fig. 8). We did not find evidence to support a relationship between OS and intra-tumor heterogeneity or wGII, both of which have previously been purported to influence prognosis^23,54^ (**Supplementary Table 16**).

We examined the relationship between molecular features and progression free survival (PFS) in 167 of the patients ascertained on the basis of being at intermediate-high risk of tumour recurrence on the basis of their Leibovich score^55^ (**Supplementary Tables 1, 15 and 17, Supplementary Figs. 7 and 8**). While *VHL* status was not associated with better PFS we observed that *KDM5C* mutation was independently associated with worse outcome (HR = 1.98, 95% CI: 1.00–3.91; **Supplementary Table 16**) and a higher incidence of necrosis (OR = 4.81, 95% CI: 1.20–19.11, **Supplementary Table 18**). Thirty-seven of the 167 patients had received iCPi therapy as a first or second line treatment and in 21 of these there was documented evidence of clinical benefit. Restricting our analysis to these 37 patients, only deletion of 6q was associated with clinical benefit (OR = 7.66, 95% CI: 1.11–52.55; **Supplementary Table 19**).

## DISCUSSION

This study, to our knowledge, represents the largest WGS analysis of primary ccRCC reported to date providing for a more comprehensive description of the genomic landscape of ccRCC. We acknowledge that there are limitations to our analysis. Specifically, our reliance on short-read sequencing and lack of transcriptomic information. Nevertheless, as well as confirming established driver genes we identify new drivers further highlighting oncogenic metabolism and epigenetic reprogramming as being central to ccRCC biology. Additionally, we validate p*TERT* mutations as drivers, thereby further substantiating telomerase dysfunction in the development of ccRCC. Mutational signature analysis provides a mechanistic basis for known lifestyle and exposure risk factors as well as potentially indirectly suggesting additional ones. While we did not identify any new mutational signatures, our analysis provides further support for tobacco smoking being a risk factor for ccRCC^56^.

The large size of our study, coupled with the standardised management protocols for ccRCC patients within the UK National Health System, has enabled us to investigate the correlation between molecular features and patient prognosis. The clinical course for many ccRCC patients with apparent same stage disease can be highly variable. Upfront identification of patients who are likely to relapse early offers the prospect of intervening preemptively to maintain remission. Furthermore, since metastatic ccRCCs are chemotherapy and radiotherapy resistant, identifying tumour sub-groups with targetable molecular dependencies has the potential to inform on biologically driven therapies.

The relationship between mutations in the major clonal driver genes and patient survival has been the subject of a number of previous studies, but findings have been inconsistent^6,7,57–63^ (**Supplementary Table 20**). While some studies^7,9^ have reported *BAP1* mutations being associated with a worse clinical outcome, other studies^58,62^ have failed to demonstrate any relationship. As previously documented^9,64^, and herein, *BAP1* mutations are strongly associated with increased grade and after adjustment we failed to show support for an independent relationship. In our study, we, however, show *VHL* mutation status was independently associated with an improved OS, consistent with a recent study^61^. *VHL* mutations are early events of ccRCC development whereas other mutated genes are acquired later therefore they might be assumed to play more of a role in disease progression. Hence it is unclear why *VHL*-positive ccRCC tumours might have a more favourable outcome than *VHL*-wildtype ccRCC. Distinct evolutionary subtypes of ccRCC have, however, been proposed that appear biologically and clinically distinct, with subtypes defined being by *VHL*-wildtype, *VHL*-monodrivers, and those with multiple clonal drivers^8^. After adjusting for *VHL* status we did not find support for an independent association between other driver mutations and survivorship. Amongst the strongest relationships we identified was between increased copy number with increased survivorship, which was independent of tumour grade, presumably reflecting tumour heterogeneity. We did not find support for the purported relationship between intra-tumor heterogeneity and prognosis^23^, however our analysis did not benefit from multi-region sampling.

Although current drug treatment paradigms for ccRCC exploit targeted therapies they are primarily not directed against any specific genomic feature. To investigate the prospect of targeting specific driver mutations we queried OncoKB^32^, which is regularly curated by an expert panel and therefore generally considered to reflect the current state of knowledge. Since other investigators have reported a higher targetable variant detection rate by applying multiple tools to annotate variants we also made use of The COSMIC Mutation Actionability in Precision Oncology resource^33^. The majority of the alterations we describe as being actionable are based on clinical evidence from other cancers or biological plausibility. As per previous reports, the majority of the targetable alterations we identified are within PI3K/mTOR pathway genes. Randomised clinical trials showing clinical benefit of the mTOR inhibitors temsirolimus and everolimus in RCC have already led to their regulatory agency approval. Other targets have not been specifically studied in the context of ccRCC, hence results cannot be interpreted as definitive proof of response prediction. Examples of drugs that might be repurposed for treating ccRCC include: Temsirolimus, which is undergoing ongoing trials as a treatment for *FBXW7*-positive solid tumours^65,66^, nilotinib for *ABL1* mutations^67^, niraparib for *BAP1* mutations^68^, Tazemetostat hydrobromide for *SMARCA4* mutated cancers^69,70^, olaparib with pembrolizumab for *ARID2*-positive melanoma^71^, and alpelisib for *PIK3CA* in ER-positive metastatic breast cancer^72,73^. An important caveat to our analysis is that the genetic profiles we derived are of a single region, which has potentially limited our ability to detect clinically important sub-clonal targetable alterations.

In many other cancers a high mutational and neoantigen burden have been linked to better overall survival and responsiveness to checkpoint inhibitors presumably reflecting native immune responsiveness^74^. In our study, there was no association between neoantigen burden and OS. In contrast there was a strong relationship between increased T-cell infiltration and better prognosis. Whilst this might seem counterintuitive, however, this finding may be explained by the poor accuracy (6%) of current HLA-affinity based neoantigen prediction algorithms^75^. Accepting these limitations, the twin observations of higher T-cell infiltration being associated with better outcome and genetically predicted immune evasion is uncommon and supports the rationale for immunotherapy.

There is interest in the prospect of population screening for RCC, given the rising incidence of the disease, the high proportion of asymptomatic individuals at diagnosis and associated high mortality rate. Our analysis supports previous work suggesting that ccRCC driver mutations often precede diagnosis by many years, if not decades^29^, information relevant to the design of any screening programme.

Although some cancers have reaped demonstrable benefits from the current genomic revolution, the same benefits have not been yet observed in RCC, and further efforts should be directed to identify the precise role of genomic tumour profiling in the clinical setting.

## Data Availability

The data supporting the findings of this study are available within the Genomics England Research Environment, a secure cloud workspace. Details on how to access data for this publication can be found at https://re-docs.genomicsengland.co.uk/pan_cancer_pub/. Additional processed aggregated data supporting the findings presented in this manuscript can be found in the Supplementary Tables. To access genomic and clinical data within this Research Environment, researchers must first apply to become a member of either the Genomics England Research Network (https://www.genomicsengland.co.uk/research/academic) or the Discovery Forum (industry partners https://www.genomicsengland.co.uk/research/research-environment). The process for joining the network is described at https://www.genomicsengland.co.uk/research/academic/join-gecip and consists of the following steps:
Your institution will need to sign a participation agreement available at https://files.genomicsengland.co.uk/documents/Genomics-England-GeCIP-Participation-Agreement-v2.0.pdf and email the signed version to gecip-help@genomicsengland.co.uk. Once you have confirmed your institution is registered and have found a domain of interest, you can apply through the online form at https://www.genomicsengland.co.uk/research/academic/join-gecip. Once your Research Portal account is created you will be able to login and track your application. Your application will be reviewed within 10 working days. Your institution will validate your affiliation. You will complete online Information Governance training and will be granted access to the Research Environment within 2 days of passing the online training. Data that has been made available to registered users include: alignments in BAM or CRAM format, annotated variant calls in VCF format, signatures assignment, tumour mutation burden, sequencing quality metrics, summary of findings that is shared with Genomic Lab Hubs, secondary clinical data as described in this paper. Further details of the types of data available (for example, mortality, hospital episode statistics and treatment data) can be found at https://re-docs.genomicsengland.co.uk/data_overview/. Germline variants can be explored in Interactive Variant Analysis Browser (see description at https://re-docs.genomicsengland.co.uk/iva_variant/). Cancer patients cohort and longitudinal clinical information on treatment and mortality can be explored with Participant Explorer (see description at https://re-docs.genomicsengland.co.uk/pxa/).

## References

[1] Bukavina L..Epidemiology of Renal Cell Carcinoma: 2022 Update.Eur. Urol. 82,529–542(2022). 36100483 10.1016/j.eururo.2022.08.019

[2] Post nephrectomy management of localized renal cell carcinoma.From risk stratification to therapeutic evidence in an evolving clinical scenario.Cancer Treat. Rev.115,102528(2023). 36905896 10.1016/j.ctrv.2023.102528

[3] Choueiri T. K..Adjuvant Pembrolizumab after Nephrectomy in Renal-Cell Carcinoma.N. Engl. J. Med.385,683–694(2021). 34407342 10.1056/NEJMoa2106391

[4] Pal S. K..Adjuvant atezolizumab versus placebo for patients with renal cell carcinoma at increased risk of recurrence following resection (IMmotion010): a multicentre, randomised, double-blind, phase 3 trial.Lancet 400,1103–1116(2022). 36099926 10.1016/S0140-6736(22)01658-0

[5] Motzer R. J..Adjuvant nivolumab plus ipilimumab versus placebo for localised renal cell carcinoma after nephrectomy (CheckMate 914): a double-blind, randomised, phase 3 trial.Lancet 401,821–832(2023). 36774933 10.1016/S0140-6736(22)02574-0 PMC10259621

[6] Kapur P..Effects on survival of BAP1 and PBRM1 mutations in sporadic clear-cell renal-cell carcinoma: a retrospective analysis with independent validation.Lancet Oncol.14,159–167(2013). 23333114 10.1016/S1470-2045(12)70584-3 PMC4674067

[7] Cancer Genome Atlas Research Network.Comprehensive molecular characterization of clear cell renal cell carcinoma.Nature 499,43–49(2013). 23792563 10.1038/nature12222 PMC3771322

[8] Turajlic S..Tracking Cancer Evolution Reveals Constrained Routes to Metastases: TRACERx Renal.Cell 173,581–594.e12(2018). 29656895 10.1016/j.cell.2018.03.057 PMC5938365

[9] Hakimi A. A..Adverse outcomes in clear cell renal cell carcinoma with mutations of 3p21 epigenetic regulators BAP1 and SETD2: a report by MSKCC and the KIRC TCGA research network.Clin. Cancer Res.19,3259–3267(2013). 23620406 10.1158/1078-0432.CCR-12-3886 PMC3708609

[10] Turnbull C..The 100 000 Genomes Project: bringing whole genome sequencing to the NHS.BMJ 361,k1687(2018). 29691228 10.1136/bmj.k1687

[11] Cornish A. J..Reference bias in the Illumina Isaac aligner.Bioinformaticsvol.36 4671–4672(2020). 10.1093/bioinformatics/btaa514 PMC7653636 32437525

[12] Nik-Zainal S..The life history of 21 breast cancers.Cell 149,994–1007(2012). 22608083 10.1016/j.cell.2012.04.023 PMC3428864

[13] Chen X..Manta: rapid detection of structural variants and indels for germline and cancer sequencing applications.Bioinformatics 32,1220–1222(2016). 26647377 10.1093/bioinformatics/btv710

[14] Layer R. M., Chiang C., Quinlan A. R.&Hall I. M. LUMPY: a probabilistic framework for structural variant discovery.Genome Biol.15,R84(2014). 24970577 10.1186/gb-2014-15-6-r84 PMC4197822

[15] Rausch T..DELLY: structural variant discovery by integrated paired-end and split-read analysis.Bioinformatics 28,i333–i339(2012). 22962449 10.1093/bioinformatics/bts378 PMC3436805

[16] Martínez-Jiménez F..A compendium of mutational cancer driver genes.Nat. Rev. Cancer 20,555–572(2020). 32778778 10.1038/s41568-020-0290-x

[17] Miao D. . Genomic correlates of response to immune checkpoint therapies in clear cell renal cell carcinoma. Science 359,801–806(2018). 29301960 10.1126/science.aan5951 PMC6035749

[18] Guo G..Frequent mutations of genes encoding ubiquitin-mediated proteolysis pathway components in clear cell renal cell carcinoma.Nat. Genet.44,17–19(2011). 22138691 10.1038/ng.1014

[19] Gerlinger M..Genomic architecture and evolution of clear cell renal cell carcinomas defined by multiregion sequencing.Nat. Genet.46,225–233(2014). 24487277 10.1038/ng.2891 PMC4636053

[20] Liu J..An Integrated TCGA Pan-Cancer Clinical Data Resource to Drive High-Quality Survival Outcome Analytics.Cell 173,400–416.e11(2018). 29625055 10.1016/j.cell.2018.02.052 PMC6066282

[21] Sato Y..Integrated molecular analysis of clear-cell renal cell carcinoma.Nat. Genet.45,860–867(2013). 23797736 10.1038/ng.2699

[22] Cheng J..Accurate proteome-wide missense variant effect prediction with AlphaMissense.Science 381,eadg7492(2023). 37733863 10.1126/science.adg7492

[23] Turajlic S..Deterministic Evolutionary Trajectories Influence Primary Tumor Growth: TRACERx Renal.Cell 173,595–610.e11(2018). 29656894 10.1016/j.cell.2018.03.043 PMC5938372

[24] Cerami E..The cBio cancer genomics portal: an open platform for exploring multidimensional cancer genomics data.Cancer Discov.2,401–404(2012). 22588877 10.1158/2159-8290.CD-12-0095 PMC3956037

[25] Hoadley K. A..Cell-of-Origin Patterns Dominate the Molecular Classification of 10,000 Tumors from 33 Types of Cancer.Cell 173,291–304.e6(2018). 10.1016/j.cell.2018.03.022 PMC5957518 29625048

[26] Andreou A..Elongin C (ELOC/TCEB1)-associated von Hippel-Lindau disease.Hum. Mol. Genet.31,2728–2737(2022). 35323939 10.1093/hmg/ddac066 PMC9402235

[27] Mularoni L., Sabarinathan R., Deu-Pons J., Gonzalez-Perez A.&López-Bigas N. OncodriveFML: a general framework to identify coding and non-coding regions with cancer driver mutations.Genome Biol.17,128(2016). 27311963 10.1186/s13059-016-0994-0 PMC4910259

[28] Zhu H..Candidate Cancer Driver Mutations in Distal Regulatory Elements and Long-Range Chromatin Interaction Networks.Mol. Cell 77,1307–1321.e10(2020). 31954095 10.1016/j.molcel.2019.12.027

[29] Mitchell T. J..Timing the Landmark Events in the Evolution of Clear Cell Renal Cell Cancer: TRACERx Renal.Cell 173,611–623.e17(2018). 29656891 10.1016/j.cell.2018.02.020 PMC5927631

[30] Vandekerkhove G..Circulating Tumor DNA Reveals Clinically Actionable Somatic Genome of Metastatic Bladder Cancer.Clin. Cancer Res.23,6487–6497(2017). 28760909 10.1158/1078-0432.CCR-17-1140

[31] Allory Y..Telomerase reverse transcriptase promoter mutations in bladder cancer: high frequency across stages, detection in urine, and lack of association with outcome.Eur. Urol. 65,360–366(2014). 24018021 10.1016/j.eururo.2013.08.052

[32] Chakravarty D..OncoKB:APrecisionOncologyKnowledgeBase.JCOPrecisOncol 2017,(2017).

[33] Tate J. G..COSMIC: the Catalogue Of Somatic Mutations In Cancer.Nucleic Acids Res.47,D941–D947(2019). 30371878 10.1093/nar/gky1015 PMC6323903

[34] Cortés-Ciriano I..Comprehensive analysis of chromothripsis in 2,658 human cancers using whole-genome sequencing.Nat. Genet.52,331–341(2020). 32025003 10.1038/s41588-019-0576-7 PMC7058534

[35] Watkins T. B. K. . Pervasive chromosomal instability and karyotype order in tumour evolution. Nature 587,126–132 (2020). 32879494 10.1038/s41586-020-2698-6 PMC7611706

[36] Mermel C. H..GISTIC2.0 facilitates sensitive and confident localization of the targets of focal somatic copy-number alteration in human cancers.Genome Biol.12,R41(2011). 21527027 10.1186/gb-2011-12-4-r41 PMC3218867

[37] Masayuki T.&Yamamoto M. The KEAP1-NRF2 System in Cancer.Front. Oncol 7,85(2017). 28523248 10.3389/fonc.2017.00085 PMC5415577

[38] Lister A..Nrf2 is overexpressed in pancreatic cancer: implications for cell proliferation and therapy.Mol. Cancer 10,37(2011). 21489257 10.1186/1476-4598-10-37 PMC3098205

[39] Jiménez-Vacas J. M..Spliceosome component SF3B1 as novel prognostic biomarker and therapeutic target for prostate cancer.Transl. Res.212,89–103(2019). 31344348 10.1016/j.trsl.2019.07.001

[40] López-Cánovas J. L..Splicing factor SF3B1 is overexpressed and implicated in the aggressiveness and survival of hepatocellular carcinoma.Cancer Lett.496,72–83(2021). 33038489 10.1016/j.canlet.2020.10.010

[41] Islam S. M. A..UncoveringnovelmutationalsignaturesbydenovoextractionwithSigProfilerExtractor.CellGenom 2,None(2022).

[42] Alexandrov L. B. . The repertoire of mutational signatures in human cancer. Nature 578, 94–101 (2020). 32025018 10.1038/s41586-020-1943-3 PMC7054213

[43] Turesky R. J..Aristolochic acid exposure in Romania and implications for renal cell carcinoma. Br. J. Cancer 114,76–80 (2016). 26657656 10.1038/bjc.2015.402 PMC4716534

[44] Salipante S. J., Scroggins S. M., Hampel H. L., Turner E. H.&Pritchard C. C. Microsatellite instability detection by next generation sequencing.Clin. Chem.60,1192–1199(2014). 24987110 10.1373/clinchem.2014.223677

[45] Davies H..HRDetect is a predictor of BRCA1 and BRCA2 deficiency based on mutational signatures.Nat. Med.23,517–525(2017). 28288110 10.1038/nm.4292 PMC5833945

[46] Leshchiner I..Comprehensive analysis of tumour initiation, spatial and temporal progression under multiple lines of treatment.bioRxiv 508127(2019)doi:10.1101/508127.

[47] Gerstung M..The evolutionary history of 2,658 cancers.Nature 578,122–128(2020). 32025013 10.1038/s41586-019-1907-7 PMC7054212

[48] Hundal J..pVAC-Seq: A genome-guided in silico approach to identifying tumor neoantigens.Genome Med.8,11(2016). 26825632 10.1186/s13073-016-0264-5 PMC4733280

[49] Golkaram M..Spatiotemporal evolution of the clear cell renal cell carcinoma microenvironment links intra-tumoral heterogeneity to immune escape.Genome Med.14,143(2022). 36536472 10.1186/s13073-022-01146-3 PMC9762114

[50] Martínez-Jiménez F..Genetic immune escape landscape in primary and metastatic cancer.Nat. Genet.55,820–831(2023). 37165135 10.1038/s41588-023-01367-1 PMC10181939

[51] Kelly A.&Trowsdale J. Genetics of antigen processing and presentation.Immunogenetics 71,161–170(2019). 30215098 10.1007/s00251-018-1082-2 PMC6394470

[52] Bentham R. . Using DNA sequencing data to quantify T cell fraction and therapy response. Nature 597,555–560(2021). 34497419 10.1038/s41586-021-03894-5

[53] Gu Y.-F..Modeling Renal Cell Carcinoma in Mice: Bap1 and Pbrm1 Inactivation Drive Tumor Grade.Cancer Discov.7,900–917(2017). 28473526 10.1158/2159-8290.CD-17-0292 PMC5540776

[54] Morris L. G. T..Pan-cancer analysis of intratumor heterogeneity as a prognostic determinant of survival.Oncotarget 7,10051–10063(2016). 26840267 10.18632/oncotarget.7067 PMC4891103

[55] Leibovich B. C..Prediction of progression after radical nephrectomy for patients with clear cell renal cell carcinoma: a stratification tool for prospective clinical trials.Cancer 97,1663–1671(2003). 12655523 10.1002/cncr.11234

[56] Dose-response relationships between cigarette smoking and kidney cancer: A systematic review and meta-analysis.Crit. Rev. Oncol. Hematol.142,86–93(2019). 31387065 10.1016/j.critrevonc.2019.07.019

[57] Ravaud A..Update on the medical treatment of metastatic renal cell carcinoma.Eur. Urol.54,315–325(2008). 18485581 10.1016/j.eururo.2008.04.056

[58] Manley B. J..Integration of Recurrent Somatic Mutations with Clinical Outcomes: A Pooled Analysis of 1049 Patients with Clear Cell Renal Cell Carcinoma.Eur Urol Focus 3,421–427(2017). 28753773 10.1016/j.euf.2016.06.015 PMC5650556

[59] Hakimi A. A., Pham C. G.&Hsieh J. J. A clear picture of renal cell carcinoma.Nature geneticsvol.45 849–850(2013). 23892664 10.1038/ng.2708

[60] Smits K. M..Genetic and epigenetic alterations in the von hippel-lindau gene: the influence on renal cancer prognosis.Clin. Cancer Res.14,782–787(2008). 18245539 10.1158/1078-0432.CCR-07-1753

[61] Patard J.-J..Absence of VHL gene alteration and high VEGF expression are associated with tumour aggressiveness and poor survival of renal-cell carcinoma.Br. J. Cancer 101,1417–1424(2009). 19755989 10.1038/sj.bjc.6605298 PMC2768461

[62] Hakimi A. A..Impact of recurrent copy number alterations and cancer gene mutations on the predictive accuracy of prognostic models in clear cell renal cell carcinoma.J. Urol.192,24–29(2014). 24518768 10.1016/j.juro.2014.01.088 PMC4146751

[63] Patard J.-J..Low CAIX expression and absence of VHL gene mutation are associated with tumor aggressiveness and poor survival of clear cell renal cell carcinoma.Int. J. Cancer 123,395–400(2008). 18464292 10.1002/ijc.23496 PMC2721857

[64] Peña-Llopis S..BAP1 loss defines a new class of renal cell carcinoma.Nat. Genet.44,751–759(2012). 22683710 10.1038/ng.2323 PMC3788680

[65] Skamene T..Canadian profiling and targeted agent utilization trial (CAPTUR/PM.1): A phase II basket precision medicine trial.J. Clin. Orthod.36,TPS12127–TPS12127(2018).

[66] Dancey J. CanadianProfilingandTargetedAgentUtilizationTrial(CAPTUR) (CAPTUR).ClinicalTrials.govhttps://clinicaltrials.gov/study/NCT03297606(2023).

[67] Blay J.-Y.&Tredan O. Adapting Treatment to the Tumor Molecular Alterations for Patients With Advanced Solid Tumors: MyOwnSpecificTreatment (MOST plus).ClinicalTrials.gov https://clinicaltrials.gov/study/NCT02029001(2022).

[68] A Trial of Niraparib in BAP1 and Other DNA Damage Response (DDR)DeficientNeoplasms(UF-STO-ETI-001).ClinicalTrials.govhttps://clinicaltrials.gov/study/NCT03207347(2023).

[69] Parsons D. W..Actionable Tumor Alterations and Treatment Protocol Enrollment of Pediatric and Young Adult Patients With Refractory Cancers in the National Cancer Institute-Children’s Oncology Group Pediatric MATCH Trial.J. Clin. Oncol.40,2224–2234(2022). 35353553 10.1200/JCO.21.02838 PMC9273376

[70] Targeted Therapy Directed by Genetic Testing in Treating Pediatric Patients With Relapsed or Refractory Advanced Solid Tumors,Non-HodgkinLymphomas,orHistiocyticDisorders(ThePediatricMATCHScreeningTrial).ClinicalTrials.govhttps://clinicaltrials.gov/study/NCT03155620(2023).

[71] Kim K. B. Phase II Study of Olaparib and Pembrolizumab in Advanced Melanoma With Homologous Recombination (HR) Mutation.ClinicalTrails.gov https://clinicaltrials.gov/study/NCT04633902(2022).

[72] Zehir A..Mutational landscape of metastatic cancer revealed from prospective clinical sequencing of 10,000 patients.Nat. Med.23,1004(2017). 10.1038/nm0817-1004c 28777785

[73] Solit D.&Stadler Z. Genomic Profiling in Cancer Patients.ClinicalTrials.gov https://clinicaltrials.gov/study/NCT01775072(2023).

[74] Wang P., Chen Y.&Wang C. Beyond Tumor Mutation Burden: Tumor Neoantigen Burden as a Biomarker for Immunotherapy and Other Types of Therapy.Front. Oncol.11,672677(2021). 33996601 10.3389/fonc.2021.672677 PMC8117238

[75] Wells D. K..Key Parameters of Tumor Epitope Immunogenicity Revealed Through a Consortium Approach Improve Neoantigen Prediction.Cell 183,818–834.e13(2020). 33038342 10.1016/j.cell.2020.09.015 PMC7652061

